# Supplementary material for: Predicting suitable habitat of the Chinese monal (Lophophorus lhuysii) using ecological niche modeling in the Qionglai Mountains, China
Source: PeerJ. 2017 Jul 5;5:e3477. doi: 10.7717/peerj.3477 (PMC5501155; doi:10.7717/peerj.3477)
Supplement: Table S2 [file peerj-05-3477-s002.docx]

**Table S2. Full set of environmental variables potentially related to habitat suitability of the Chinese monal**

| Group | Predictor code | Description | Units | Original resolution |
| --- | --- | --- | --- | --- |
| Climatic data | bioclim1 | Annual mean temperature | °C | 1 km |
|  | bioclim2 | Mean diurnal range (max temp-min temp) | °C | 1 km |
|  | bioclim3 | Isothermality (bioclim2/bioclim7) | ratio | 1 km |
|  | bioclim4 | Temperature seasonality | standard deviation | 1 km |
|  | bioclim5 | Max temperature of warmest month | °C | 1 km |
|  | bioclim6 | Min temperature of coldest month | °C | 1 km |
|  | bioclim7 | Temperature annual range (bioclim5-bioclim6) | °C | 1 km |
|  | bioclim8 | Mean temperature of wettest quarter | °C | 1 km |
|  | bioclim9 | Mean temperature of driest quarter | °C | 1 km |
|  | bioclim10 | Mean temperature of warmest quarter | °C | 1 km |
|  | bioclim11 | Mean temperature of coldest quarter | °C | 1 km |
|  | bioclim12 | Annual precipitation | mm | 1 km |
|  | bioclim13 | Precipitation of wettest month | mm | 1 km |
|  | bioclim14 | Precipitation of driest month | mm | 1 km |
|  | bioclim15 | Precipitation seasonality | coefficient of variation | 1 km |
|  | bioclim16 | Precipitation of wettest quarter | mm | 1 km |
|  | bioclim17 | Precipitation of driest quarter | mm | 1 km |
|  | bioclim18 | Precipitation of warmest quarter | mm | 1 km |
|  | bioclim19 | Precipitation of coldest quarter | mm | 1 km |
| Phenological metrics | evi maximum | Annual maximum EVI | continuous, 0-1 | 250 m |
|  | evi base level | Average between the minimum values at the start and end of each cycle | continuous, 0-1 | 250 m |
|  | evi mean | Annual mean EVI | continuous, 0-1 | 250 m |
|  | evi sum | Annual sum EVI | continuous | 250 m |
|  | evi std | Standard deviation of annual mean EVI | standard deviation | 250 m |
|  | evi cv | Coefficient of variation of annual mean EVI | coefficient of variation | 250 m |
|  | evi amplitude | EVI annual range (max EVI-min EVI) | continuous | 250 m |
|  | season start | Date when EVI increase to half of annual amplitude at the start of a phenology cycle | #day, 1-365 | 250 m |
|  | season end | Date when EVI decrease to half of annual amplitude at the end of a phenology cycle | #day, 1-365 | 250 m |
|  | season middle | Date of the middle of the season, when EVI reach the peak value | #day, 1-365 | 250 m |
|  | season length | Mean duration of phenology seasons (from the start to the end of the season) | day | 250 m |
|  | large integral | Integral area under the smoothed EVI curve between the start and the end of the season | continuous | 250 m |
|  | small integral | Integral area under the smoothed EVI curve and above the base level, between the start and the end of the season | continuous | 250 m |
|  | left derivative | Increase rate of EVI, calculated as the slope across the 20% and 80% level points of the season starting | continuous | 250 m |
|  | right derivative | Decrease rate of EVI, calculated as the slope across the 80% and 20% level points of the season ending | continuous | 250 m |
| Vegetation | landcover | Land cover types classified as IGBP system | categorical, 17 land types | 500 m |
| Topographical attributes | elevation | Elevation above sea level | m | 30 m |
|  | aspect | Direction of slope, calculated as absolute value of actual direction minus 180° | ° | 30 m |
|  | slope | Gradient of slope | ° | 30 m |
|  | d_river | Euclidean distance to the nearest perennial river | m | Generated |
| Human impacts | HII | Human Influence Index, generated combining human population pressure, accessibility, and land use and infrastructure | continuous, 0-64 | 1 km |
|  | d_resident | Euclidean distance to the nearest residential location | m | Generated |
|  | d_road | Euclidean distance to the nearest road | m | Generated |
