# Supplementary material for: Predicting suitable habitat of the Chinese monal (Lophophorus lhuysii) using ecological niche modeling in the Qionglai Mountains, China
Source: PeerJ. 2017 Jul 5;5:e3477. doi: 10.7717/peerj.3477 (PMC5501155; doi:10.7717/peerj.3477)
Supplement: Table S3 — The optimized selection of environmental variables started with all 42 variables and stepwise removed the variables with high correlation with others (Pearson’s correlation coefficients >0.7) and low contribution (<1%). Performance of each model was estimated by the sample-size-corrected Akaike information criterion (AICc). Model 7 had the lowest AICc value, indicating that the seven variables included in this model represents the most appropriate model complexity. [file peerj-05-3477-s003.docx]

Table S3. Performance of models with different sets of environmental variables. The optimized selection of environmental variables started with all 42 variables and stepwise removed the variables with high correlation with others (Pearson’s correlation coefficients ﹥｜0.7｜) and low contribution (﹤1%). Performance of each model was estimated by the sample-size-corrected Akaike information criterion (AICc). Model 7 had the lowest AICc value, indicating that the seven variables included in this model represents the most appropriate model complexity.

| Model | variables | parameters | AICc |
| --- | --- | --- | --- |
| 1 | 42 | 76 | - |
| 2 | 15 | 45 | 1644.155 |
| 3 | 9 | 32 | 1494.262 |
| 4 | 9 | 32 | 1494.262 |
| 5 | 8 | 32 | 1494.438 |
| 6 | 8 | 32 | 1494.438 |
| 7 | 7 | 25 | 1453.580 |
