# Supplementary material for: Predicting suitable habitat of the Chinese monal (Lophophorus lhuysii) using ecological niche modeling in the Qionglai Mountains, China
Source: PeerJ. 2017 Jul 5;5:e3477. doi: 10.7717/peerj.3477 (PMC5501155; doi:10.7717/peerj.3477)
Supplement: Table S4 — The optimized selection of environmental variables simplified variables in a stepwise fashion: model 1 was built with all set of 42 variables, and 27 variables were removed in model 2 as they had low contribution (<1%) or high correlation (>0.7 or <0.7) with the highest contribution variable (bioclim17), and 6 variables were removed in model 3 as their low contribution or high correlation with the second high contribution variable (bioclim5), finally seven models were produced after repeating this process. Model 7 had the lowest AICc value (Table S3), indicating this model included the variables represent the most appreciate complexity. Therefore, these seven variables were selected as predictors for building the final MaxEnt model for Chinese monal habitat. Note: con, percent contribution; cor, Pearson’s correlation coefficient. Check Table S3 for meaning and information of each environmental variable. [file peerj-05-3477-s004.docx]

Table S4. Process of the optimized environmental variable selection. The optimized selection of environmental variables simplified variables in a stepwise fashion: model 1 was built with all set of 42 variables, and 27 variables were removed in model 2 as they had low contribution (﹤1%) or high correlation (﹥0.7 or ﹤0.7) with the highest contribution variable (bioclim17), and 6 variables were removed in model 3 as their low contribution or high correlation with the second high contribution variable (bioclim5), finally seven models were produced after repeating this process. Model 7 had the lowest AICc value (Table S3), indicating this model included the variables represent the most appreciate complexity. Therefore, these seven variables were selected as predictors for building the final MaxEnt model for Chinese monal habitat. Note: con, percent contribution; cor, Pearson’s correlation coefficient. Check Table S2 for meaning and information of each environmental variable.

|  | con | cor | con | cor | con | cor | con | cor | con | cor | con | cor | con | cor |
| --- | --- | --- | --- | --- | --- | --- | --- | --- | --- | --- | --- | --- | --- | --- |
| Model | 1 | 1 | 2 | 2 | 3 | 3 | 4 | 4 | 5 | 5 | 6 | 6 | 7 | 7 |
| aspect | 1.1 | 0.0 | 0.8 | - | - | - | - | - | - | - | - | - | - | - |
| bioclim1 | 3.7 | 0.5 | 4.7 | 1.0 | - | - | - | - | - | - | - | - | - | - |
| bioclim10 | 0.1 | - | - | - | - | - | - | - | - | - | - | - | - | - |
| bioclim11 | 0.1 | - | - | - | - | - | - | - | - | - | - | - | - | - |
| bioclim12 | 3.0 | 1.0 | - | - | - | - | - | - | - | - | - | - | - | - |
| bioclim13 | 0.8 | - | - | - | - | - | - | - | - | - | - | - | - | - |
| bioclim14 | 0.1 | - | - | - | - | - | - | - | - | - | - | - | - | - |
| bioclim15 | 0.8 | - | - | - | - | - | - | - | - | - | - | - | - | - |
| bioclim16 | 0.0 | - | - | - | - | - | - | - | - | - | - | - | - | - |
| bioclim17 | 25.0 | 1.0 | 29.3 | 0.5 | 35.6 | -0.1 | 36.5 | 0.3 | 37.1 | -0.5 | 33.4 | 0.1 | 35.1 | -0.1 |
| bioclim18 | 0.0 | - | - | - | - | - | - | - | - | - | - | - | - | - |
| bioclim19 | 1.5 | 1.0 | - | - | - | - | - | - | - | - | - | - | - | - |
| bioclim2 | 2.2 | -0.6 | 5.0 | -0.8 | - | - | - | - | - | - | - | - | - | - |
| bioclim3 | 3.5 | -0.7 | - | - | - | - | - | - | - | - | - | - | - | - |
| bioclim4 | 0.0 | - | - | - | - | - | - | - | - | - | - | - | - | - |
| bioclim5 | 22.1 | 0.5 | 17.2 | 1.0 | 27.5 | -0.5 | 31.0 | 0.6 | 30.4 | -0.4 | 28.8 | 0.6 | 25.9 | -0.4 |
| bioclim6 | 2.0 | 0.5 | 3.0 | 1.0 | - | - | - | - | - | - | - | - | - | - |
| bioclim7 | 4.0 | -0.5 | 5.1 | -0.7 | - | - | - | - | - | - | - | - | - | - |
| bioclim8 | 0.1 | - | - | - | - | - | - | - | - | - | - | - | - | - |
| bioclim9 | 0.1 | - | - | - | - | - | - | - | - | - | - | - | - | - |
| d_resident | 7.8 | -0.1 | 14.6 | -0.5 | 16.2 | 1.0 | 10.9 | -0.4 | 12.9 | 0.2 | 15.4 | -0.5 | 19.2 | 0.5 |
| d_river | 0.4 | - | - | - | - | - | - | - | - | - | - | - | - | - |
| d_road | 1.0 | -0.1 | 2.2 | -0.4 | 1.0 | 0.5 | 2.4 | -0.3 | 1.0 | 0.2 | 1.7 | -0.4 | 2.7 | 1.0 |
| elevation | 1.8 | -0.6 | 2.8 | -1.0 | - | - | - | - | - | - | - | - | - | - |
| season end | 0.1 | - | - | - | - | - | - | - | - | - | - | - | - | - |
| evi amplitude | 0.6 | - | - | - | - | - | - | - | - | - | - | - | - | - |
| evi cv | 0.6 | - | - | - | - | - | - | - | - | - | - | - | - | - |
| evi maximum | 2.1 | 0.1 | 2.8 | 0.6 | 3.3 | -0.5 | 3.0 | 0.7 | 2.5 | -0.2 | 2.5 | 1.0 | 3.4 | -0.4 |
| evi mean | 0.1 | - | - | - | - | - | - | - | - | - | - | - | - | - |
| evi base level | 6.6 | 0.3 | 7.4 | 0.6 | 8.9 | -0.4 | 9.6 | 1.0 | 12.1 | -0.2 | 10.3 | 0.7 | 9.9 | -0.3 |
| evi std | 0.4 | - | - | - | - | - | - | - | - | - | - | - | - | - |
| evi sum | 0.1 | - | - | - | - | - | - | - | - | - | - | - | - | - |
| HII | 0.8 | - | - | - | - | - | - | - | - | - | - | - | - | - |
| landcover | 0.3 | - | - | - | - | - | - | - | - | - | - | - | - | - |
| large integral | 1.1 | 0.1 | 1.2 | 0.7 | 2.7 | -0.5 | 0.6 | - | - | - | - | - | - | - |
| left derivative | 0.4 | - | - | - | - | - | - | - | - | - | - | - | - | - |
| season length | 0.3 | - | - | - | - | - | - | - | - | - | - | - | - | - |
| season middle | 0.2 | - | - | - | - | - | - | - | - | - | - | - | - | - |
| right derivative | 0.6 | - | - | - | - | - | - | - | - | - | - | - | - | - |
| slope | 2.7 | -0.5 | 2.8 | -0.4 | 3.7 | 0.2 | 3.4 | -0.2 | 2.8 | 1.0 | 5.7 | -0.2 | 3.9 | 0.2 |
| small integral | 1.4 | -0.1 | 1.0 | 0.5 | 1.1 | -0.4 | 2.5 | 0.2 | 1.2 | 0.0 | 2.2 | 0.7 | - | - |
| season start | 0.5 | - | - | - | - | - | - | - | - | - | - | - | - | - |
